# The Influence of Sheet Tilting on Forming Quality in Single Point Incremental Forming

**DOI:** 10.3390/ma14143907

**Published:** 2021-07-13

**Authors:** Hu Zhu, Yang Wang, Yibo Liu, Dongwon Jung

**Affiliations:** 1 College of Mechanical and Electrical Engineering, Shenyang Aerospace University, Shenyang 110136, China; 18940083334@163.com; 2 Science and Technology Training Center, Guidaojiaotong Polytechnic Institute, Shenyang 110023, China; 15140152358@163.com; 3 Department of Mechanical Engineering, Jeju National University, Jeju-Do 63243, Korea

**Keywords:** incremental forming, single point forming, sheet tilting, forming quality

## Abstract

In the CNC incremental forming process, the sheet tilting method can be used to realize the non-fracture forming of a surface with large forming angles. However, the forming effect of the formed part will have big differences when the inclined angle of the sheet is different. Therefore, four different tilted sheets with inclined angles of 15°, 20°, 25°, and 30° were used to study the influence of sheet tilting on forming quality by using 1060 Aluminum sheet as the forming sheet in single point CNC incremental forming. First, the influence of four different inclined angles of the sheet on the overall thickness distribution, plastic strain, and material flow of the formed part was studied by using numerical simulation. Then, the influence of four different inclined angles of sheets on the profile accuracy and thickness thinning rate of the formed part was studied through single point incremental forming experiments. The research results show that sheet tilting has little effect on the profile accuracy of the formed part, but has a great influence on the material flow, plastic strain, and thickness distribution.

## 1. Introduction

The sheet metal CNC incremental forming process is a new, dieless forming technology developed recently [1] that combines plastic forming technology with layered manufacturing technology, in which the forming tool moves along the pre-programed forming toolpath under the control of numerical control equipment and extrudes the sheet metal point by point to form the required sheet metal parts gradually and without expensive dies [2]. Therefore, this technology has a good application prospect in the production of many varieties and few batches [3].

Although CNC incremental forming technology has developed rapidly in recent years, it has had some difficulty in the forming of sheet metal parts with larger forming angles, such as vertical-wall parts by single-stage forming [4], which always restricts the wide application and development of this technology [5,6]. The horizontal sheets are usually adopted in the traditional sheet metal CNC incremental forming technology. According to the thinning law of sheet metal thickness [7], the sheet metal will deform sharply during the forming process which will cause the sheet to crack when the forming angle is large. 

At present, the multistage forming technology is used to solve the forming of the sheet metal parts with larger forming angles by most scholars [8]. For example, Kim and Yang [9] reported that two-stage forming can improve formability and increase thickness uniformity compared to the single-stage forming. Hirt et al. [10] proposed a multistage forming strategy with increasing angle and realized the forming of square cone. Gupta et al. [11] experimentally evaluated various multistage strategies and successfully manufactured a complex C-channel fixture designed for aircraft vibration testing. Zhou et al. [12] presented three forming toolpath strategies (i.e., parallel line type tool path, curve-type tool path, and straight line type path with a variable angle) to form vertical wall shell parts and found that the uniform wall thickness of the vertical wall cylinder parts can be obtained via the parallel linear tool path. Liu et al. [13] proposed three kinds of multistage forming toolpath strategies (i.e., incremental part diameter, incremental draw angle, and incremental part height and draw angle) and evaluated these strategies and their combinations according to formability and material flow. They found that the strategic combination A+B is the optimal way to achieve the desired forming quality. 

Skjoedt et al. [14] proposed a multistage forming strategy based on DUDD and DDDU and formed a cylindrical cup with the step features occured at the bottom. Duflou et al. [15] formed a cone by using an angle increasing multistage forming strategy and found that the step features had been occurred in the forming process. Liu et al. [16] proposed an open-loop analysis model based on thickness strain and established a closed-loop multistage design method. Dai et al. [17] reduced the error of the stepped feature by using path compensated three-stage forming. Nirala and Agrawal [18] proposed an incremental forming toolpath based on fractal geometry, which can improve the strength of sheet metal. Li et al. [19,20] pointed out that the stepped feature is the main factor affecting its accuracy and proposed a prediction model. Lingam et al. [21] pointed out that rigid body motion (RBM) exists in multistage incremental forming and provided a method to predict rigid body motion. Malhotra et al. [22] proposed a method to reduce step feature based on an IO-OI hybrid path. Ndip Agbor et al. [23] proposed a RBM prediction method based on the contact spot model between the forming tool head and the sheet metal. Mostafanezhad et al. [24] studied the effects of the forming angle, tool diameter, and sheet thickness on the sheet thinning rate as well as the forming force in single-point incremental forming and found that the forming angle has the greatest effect on the sheet thinning rate.

Xiao et al. [25] analyzed the influence of forming parameters on thickness thinning and limiting forming angle by using the response surface method as well as neural network and optimized the forming parameters through a genetic algorithm. Li et al. [26] compared three kinds of multi-pass forming methods (parallel line, variable angle, and stretch-bending auxiliary) in the incremental forming. The results showed that these three methods could improve the thickness distribution as well as the geometric error of taper parts; the forming effect was better than that of the variable angle method. Cao et al. [27] proposed a thickness prediction method for multistage incremental forming. Li et al. [28] studied the influence of the number of forming passes on the forming quality and found that the minimum thickness of the formed part increased with the increase in the number of forming stages, but the springback was relatively increased at the same time. 

Although the above-mentioned scholars have improved the thickness distribution of formed parts with large forming angles through the multistage forming method, these studies are based on the horizontal sheet and the deformation mode of the sheet metal was not changed.

In the CNC incremental forming process, the sheet postures were determined by the extrusion motion of the forming tool. The forming angles were also changed in the forming process when the horizontal sheet was changed into the inclined sheet via the extrusion motion of the forming tool. The thickness of the formed part is determined by the forming angles during the forming process, which provides the possibility to solve the above problems; scholars have performed some research, as follows.

Vanhove et al. [29] studied the method for forming the elliptical cone parts with different slopes between the left and right sides by tilting the sheet in a certain direction. Tanaka et al. [30] used the inclined plane to cut the model to generate the toolpath to obtain the inclined sheet and realized the non-rupture forming of the formed part with large forming angles. Zhu and Li [31] obtained the inclined sheet in different orientations directly by controlling the squeezing movement of the forming tool and realized the non-rupture forming of the sheet metal part, including straight wall. The above-mentioned scholars have proved the feasibility of the sheet tilting method in the CNC incremental forming process through their own research, but the influence of sheet tilting on forming quality was not studied. In order to homogenize the thickness of the formed part, Zhu et al. [32] optimized a set of inclined toolpaths that were parallel to each other through a genetic algorithm and adjusted the inclination of the sheet using the optimized toolpaths. Zhu et al. [33] optimized a set of sheets with different inclined angles via the particle swarm algorithm, so that the formed part with uniform thickness can be obtained. 

The above-mentioned scholars only used the optimized sheet postures to conduct experiments in a single-forming process, which proved that their respective studies could make the thickness of the formed parts more uniform, but they did not conduct multiple comparison tests by using sheets with different inclination angles. Therefore, the influence of sheet tilting on the forming quality has not been studied. 

Sheet metal can be inclined to a certain extent via the extrusion motion of the forming tool in the CNC incremental forming process and then the surface with the large forming angles can be formed without fracturing. H owever, the forming effect of the formed parts will make a big difference when the inclined angles of the sheet metal are different.

In this paper, the influence of sheet tilting on the forming quality was studied using four different sheets with inclined angles of $15^{\circ }$, $20^{\circ }$, $25^{\circ }$, and $30^{\circ }$ based on numerical simulation and forming experiments performed by using 1060 Aluminum sheet as the forming sheet.

## 2. The General Method

**Figure 1:**
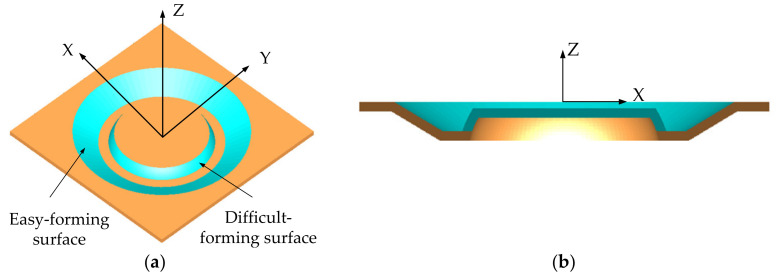
Case model: (**a**) isometric side view and (**b**) cutaway view.

**Figure 2:**
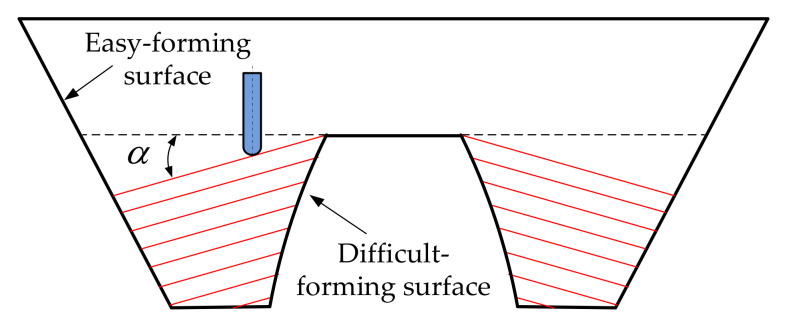
Schematic diagram of inclined sheet.

In order to study the influence of sheet tilting on forming quality in single-point incremental forming and eliminate the interference of model contour on forming quality in forming experiment, the regular circular contour model, as shown in Figure 1, was selected as an example to study the influence of sheet tilting on forming quality through finite element numerical simulation and the incremental forming experiment. The easy-forming surface is a single curvature surface with a forming angle of $30^{\circ }$ and the difficult-forming surface is a surface with large forming angles and gradual curvature; the maximum forming angle is $75^{\circ }$. For the sheet metal part model, the optional range of the inclined angle α of the sheet in the incremental forming experiment is $10^{\circ }\leq \alpha \leq 35^{\circ }$, according to the calculation method [33] for sheet optional inclined angles studied by our research group; the diagram is shown in Figure 2.

The inclined angle α of the sheet with equal difference extents were selected as as $15^{\circ }$, $20^{\circ }$, $25^{\circ }$, and $30^{\circ }$ respectively, within the allowable inclined range of sheets in this paper, so that the forming angles and their differences in four numerical simulations and forming experiments were also changed with equal difference extents; additionally, the influence on the forming quality using different inclined angles of the sheet was studied. Firstly, the influence of sheet tilting on the thickness distribution, plastic strain, and material flow of the formed part was studied by using a finite element numerical simulation with the ANSYS/LS-DYNA software. Then, the influence of sheet tilting on the profile accuracy and thickness reduction rate of the formed parts was studied through the incremental forming experiments.

**Figure 3:**
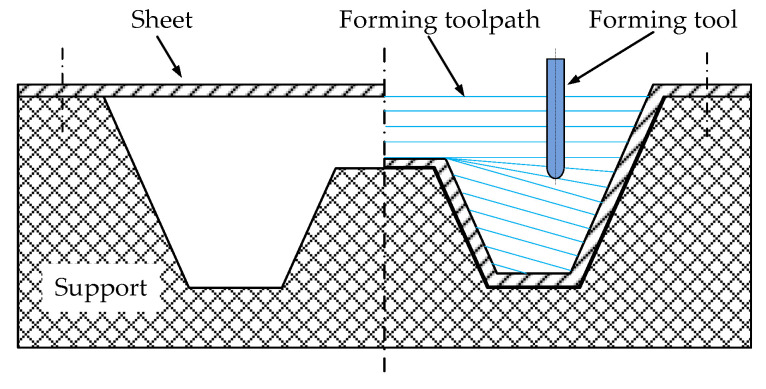
Schematic diagram of the forming process.

The numerical simulations and forming experiments were conducted via three-axis CNC incremental forming (as shown in Figure 3), namely, the sheet was fixed on the support with bolts, the support was placed on the workbench of the three-axis CNC machining center, and the spindle of the machine tool drove the forming tool for forming processing. The 1060 Aluminum has good formability, which is usually taken as an example material in scientific research by many scholars in CNC incremental forming. In order to eliminate the interference of material factors on forming quality and to reflect the influence of sheet posture on forming quality to the maximum extent, the 1060 Aluminum sheet was taken as the forming sheet in this study. In addition, the hemispherical tool that is the most commonly used in the actual production and scientific experiment of CNC incremental forming was selected as the forming tool. 

## 3. Numerical Simulation

### 3.1. Parameter Setting of the Finite Element Numerical Simulation

**Figure 4:**
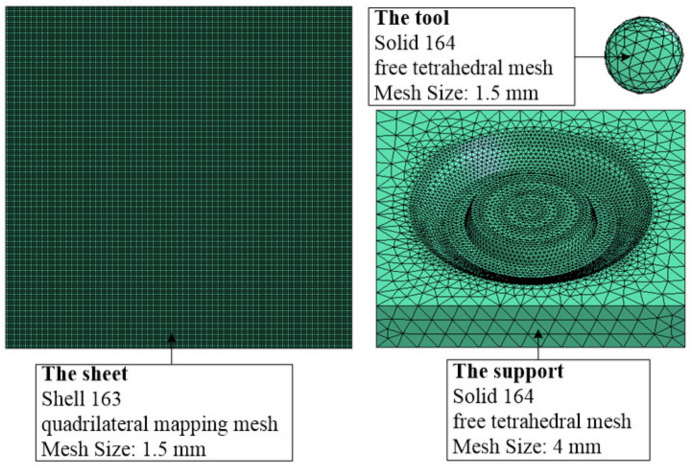
Mesh model for finite element analysis.

The parameters for the finite element analysis were set as follows: for the sheet, the material was defined as a 1060 Aluminum sheet with a thickness of 0.88 mm, the element type selected was a “SHELL 163” (a thin shell element), the algorithm type selected was “Belytschko-Wong” for this element, and the mesh type selected was a quadrilateral mapping mesh of 1.5 mm size. The support was defined as rigid and its material was set as high-speed steel (W6Mo5Cr4V2). The element type and mesh type were set as a “SOLID 164” hexahedron element and a tetrahedral free mesh of 4 mm size, respectively. The forming tool was also defined as rigid and was set as a hemispherical tool with a diameter of 10 mm, and its material was set as high speed steel (W6Mo5Cr4V2). The element type and mesh type were set as a “SOLID 164” hexahedron element and a tetrahedral free mesh of 1.5 mm size, respectively. The finite element analysis mesh model is shown as Figure 4. The mechanical properties of each material is shown in Table 1.

**Figure 5:**
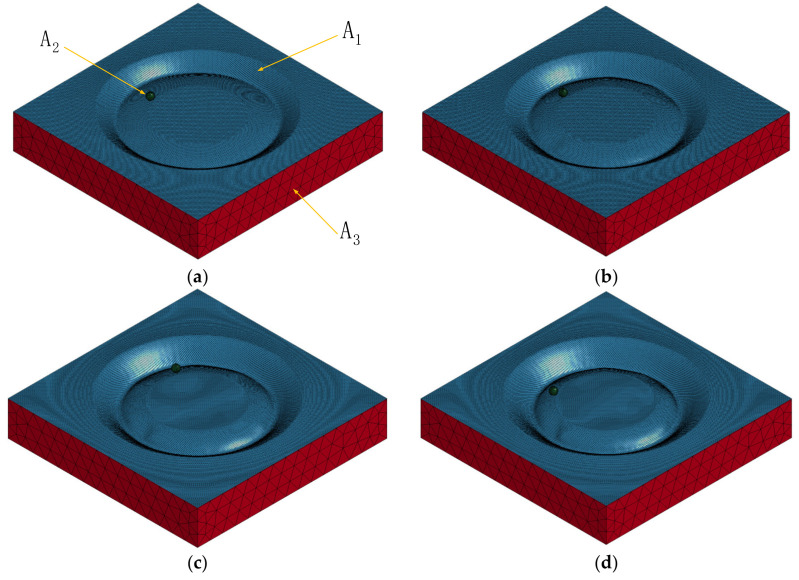
The process of numerical simulation: (**a**) the inclined angle of the sheet is $15^{\circ }$, (**b**) the inclined angle of the sheet is $20^{\circ }$, (**c**) the inclined angle of the sheet is $25^{\circ }$, and (**d**) the inclined angle of the sheet is $30^{\circ }$.

The finite element numerical simulation process is shown in Figure 5: Figure 5a–d shows the finite element numerical simulation process, respectively, when the inclined angles of the sheet were $15^{\circ }$, $20^{\circ }$, $25^{\circ }$, and $30^{\circ }$, in which the blue deformation area ($A_{1}$) represents the sheet metal, the green sphere ($A_{2}$) represents the forming tool, and the red area ($A_{3}$) represents the support.

### 3.2. Analysis of the Finite Element Numerical Simulation Results

#### 3.2.1. Thickness Distribution

**Figure 6:**
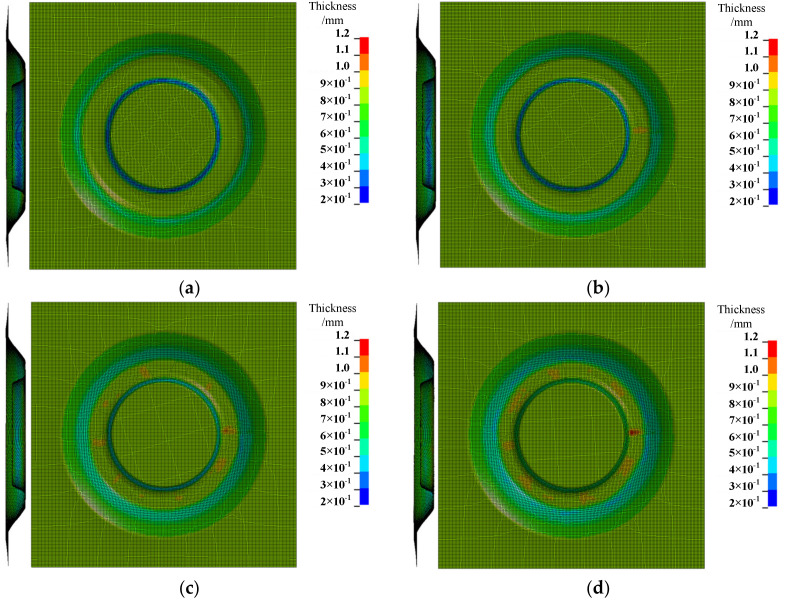
The thickness distribution cloud map: (**a**) the inclined angle of the sheet is $15^{\circ }$, (**b**) the inclined angle of the sheet is $20^{\circ }$, (**c**) the inclined angle of the sheet is $25^{\circ }$, and (**d**) the inclined angle of sheet is $30^{\circ }$.

The results shown in Figure 6 compare the thickness distribution cloud map under the sheet of four different inclined angles (the inclined angles were $15^{\circ }$, $20^{\circ }$, $25^{\circ }$, and $30^{\circ }$, respectively).

According to the thickness distribution cloud map, when the inclined angles of the sheets were $15^{\circ }$, $20^{\circ }$, $25^{\circ }$, and $30^{\circ }$, respectively, the maximum thickness of the formed parts was 0.89 mm, 0.97 mm, 1.03 mm, and 1.19 mm, respectively; the minimum thickness was 0.22 mm, 0.24 mm, 0.35 mm, and 0.39 mm, respectively. At this time, the maximum difference of the thickness of the formed parts was 0.67 mm, 0.73 mm, 0.68 mm, and 0.80 mm.

The results show that the maximum thickness of the formed part was distributed at the bottom of the forming area under the four different inclined angles of the sheet posture. The minimum thickness of the formed part was distributed on the difficult-forming surface when the inclined angles of the sheet postures were $15^{\circ }$, $20^{\circ }$ and $25^{\circ }$; the minimum thickness of the formed part was distributed at the bottom of the easy-forming surface when the inclined angles of the sheet posture was $30^{\circ }$. The thickness of the easy-forming surface decreased with the increase of the inclined angle of the sheet posture, while the thickness of the difficult-forming surface increased with the increase of the inclined angle of the sheet posture.

It can be known from the research contents of Zhu and Li [31] that the forming angles on the difficult-forming surface decreases while the forming angles on the easy-forming surface increases when the sheet postures are inclined from the difficult-forming surface to the easy-forming surface, so the thinning degree on the easy-forming surface gradually increases and the thinning degree on the difficult-forming surface gradually decreases.

According to our previous research [33], the forming angles on the easy-forming surface is equal to the sum of the inclined angle of the sheet posture and the initial forming angle; the forming angles on the difficult-forming surface is equal to the difference between the inclined angle of the sheet posture and the initial forming angle after the adjustment of the sheet posture. Therefore, the variation of the forming angles with the inclined angles of the sheet posture is shown in Table 2.

When the inclined angle of the sheet was $15^{\circ }$, the maximum forming angle on the difficult-forming surface was far greater than that of the easy-forming surface; however, when the inclined angles of the sheet were $20^{\circ }$ and $25^{\circ }$, the maximum forming angle on the difficult-forming surface was almost the same as that of the easy-forming surface. Therefore, the minimum thickness of the sheet occurred on the difficult-forming surface.

When the inclined angle of sheet was $30^{\circ }$, the forming angle on the easy-forming surface was far greater than the maximum forming angle on the difficult-forming surface. Therefore, the minimum thickness of the sheet occurred on the easy-forming surface. The thickness of the easy-forming surface gradually decreased with the continuous forming process and, finally, the minimum thickness occurred on the bottom of the easy-forming surface.

#### 3.2.2. Plastic Strain

**Figure 7:**
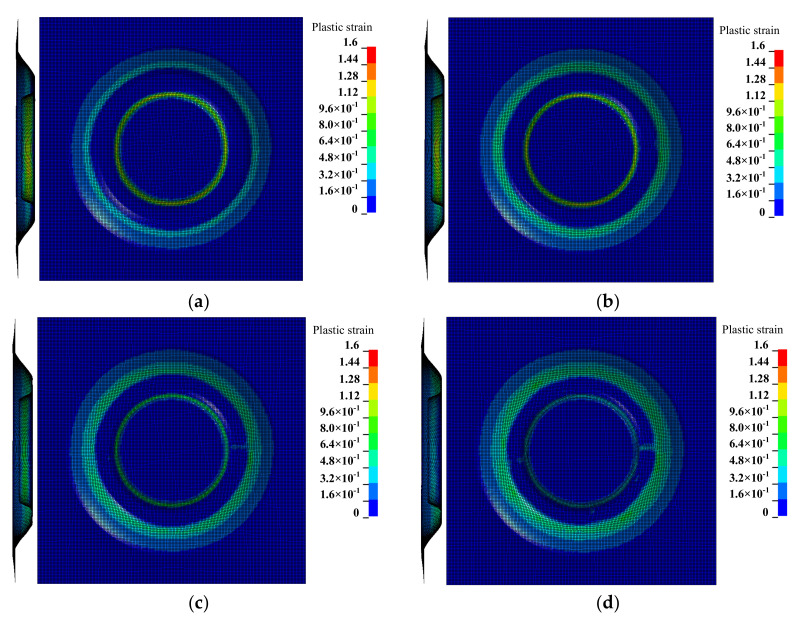
The plastic strain distribution cloud map: (**a**) the inclined angle of the sheet is $15^{\circ }$, (**b**) the inclined angle of the sheet is $20^{\circ }$, (**c**) the inclined angle of the sheet is $25^{\circ }$, and (**d**) the inclined angle of the sheet is $30^{\circ }$.

**Figure 8:**
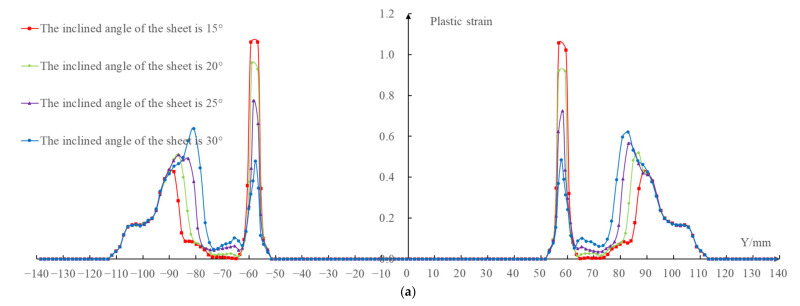

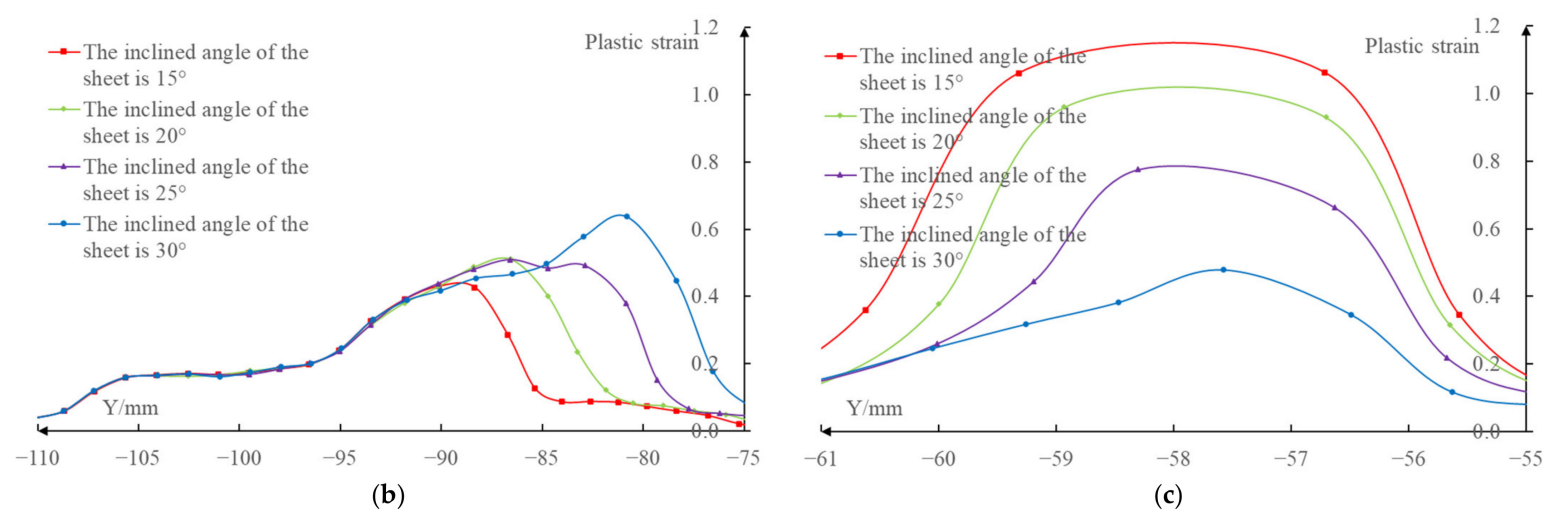
Plastic strain contrast curve: (**a**) X = 0 section, (**b**) easy-forming surface, and (**c**) difficult-forming surface.

The results shown in Figure 7 compare the plastic strain distribution cloud map under four different inclined angles of the sheet (the inclined angles were $15^{\circ }$, $20^{\circ }$, $25^{\circ }$, and $30^{\circ }$, respectively). The overall comparison curve was shown in Figure 8a after the plastic strain values of each node on the *X* = 0 section were extracted. Because the change of plastic strain on the left and right sides of the forming area were basically consistent, in order to clearly compare the effects under the four different inclined angles of the sheet on the plastic strain in the forming area, the plastic strain values of each node on the easy-forming surface and the difficult-forming surface at the *X* = 0 section in the left-forming area were separately extracted; the comparison curves are shown in Figure 8b,c.

It can be seen from the data in the figure that the plastic strain value on the easy-forming surface (−110 mm≤Y≤-78 mm) increased with the increase of the inclined angle of the sheet, while the plastic strain value on the difficult-forming (−61 mm≤Y≤−55 mm) surface decreased with the increase of the inclined angle of sheet for the left-forming area. The results show that the deformation degree and thickness reduction degree of the easy-forming surface increased with the increase of the inclined angle of the sheet, while the deformation degree and thickness reduction degree on the difficult-forming surface decreased with the increase of the inclined angle of the sheet, which was consistent with the thickness distribution cloud map. The changing rule of the plastic strain in the right-forming area was consistent with the left-forming area.

The maximum plastic strain value appeared on the difficult-forming surface when the inclined angles of the sheet were $15^{\circ }$, $20^{\circ }$, $25^{\circ }$, namely, the minimum thickness was distributed on the difficult-forming surface. The maximum plastic strain value appeared on the easy-forming surface when the inclined angle of the sheet was $30^{\circ }$, i.e., the minimum thickness was distributed on the easy-forming surface. The changing law of the plastic strain was consistent with the changing law of the thickness distribution. The reason for the difference in the maximum plastic strain distribution was consistent with that of the thickness distribution, i.e., the forming angle on the easy-forming surface increased gradually with the increase of the inclined angle of the sheet postures. The forming angle on the easy-forming surface was far greater than that of the difficult-forming surface when the inclined angle of the sheet reached $30^{\circ }$; the deformation degree of the sheet metal on the easy-forming surface was greater at this time, i.e., the maximum plastic strain was distributed on the easy-forming surface.

#### 3.2.3. Material Flow

**Figure 9:**
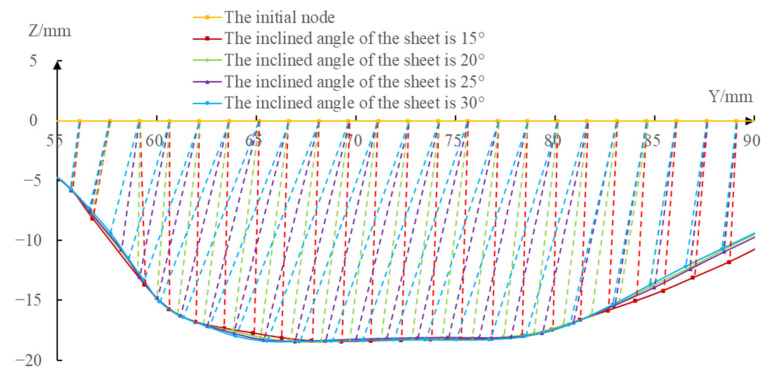
Schematic diagram of the node flow curves.

First, extract the initial coordinate positions of the same node in the different inclined angles of the sheet (the initial coordinate positions of the nodes under the different inclined angles of the sheet were consistent) and the coordinate positions after forming, respectively, in the area with obvious differences in plastic strain (-90 mm≤Y≤−55 mm and 55 mm≤Y≤90 mm) under the four different inclined angles of the sheet on the *X* = 0 section. Then, make the four node distribution curves (shown as the solid line in Figure 9) by successively connecting them using the smooth curves. Finally, obtain the node flow curves under the different inclined angles of the sheet by connecting the coordinate positions before and after the deformation of each node (shown as the dashed line in Figure 9).

It can be seen from Figure 9 that although the distribution curve trend of the node after deformation under the different inclined angles of the sheet was consistent in the whole forming area, there were obvious differences in the node flow curves, i.e., the flow direction and the flow distance of the same node under the different inclined angles of the sheet were obviously different. The flow curves of the same node under the different inclined angles of the sheet were fan-shaped and the order from left to right of the inclined angles were $15^{\circ }$, $25^{\circ }$, $20^{\circ }$ and $15^{\circ }$. When 55 mm≤Y≤60 mm, the size of the fan-shaped area was smaller, the flow direction and distance of the nodes under the different inclined angles of the sheet tended to be consistent. When 60 mm≤Y≤85 mm, the size of the fan-shaped area was larger, the flow direction and distance of the nodes under the different inclined angles of the sheet had a great difference. When 85 mm≤Y≤90 mm, the size of the fan-shaped area was smaller and the flow direction and distance of the nodes under the different inclined angles of sheet tended to be consistent.

**Figure 10:**
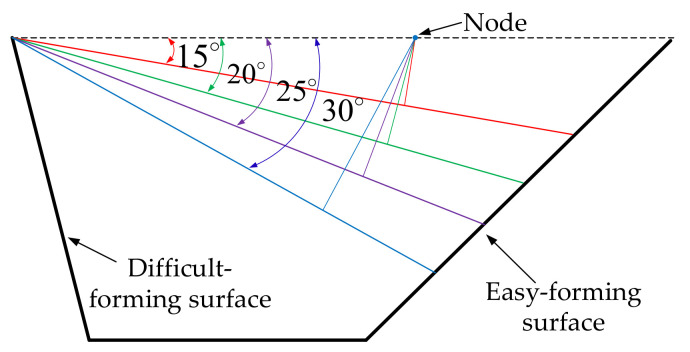
The normal direction of sheet postures.

According to the researches of Bambach [34] and Duflou et al. [15], the material point moves along the normal direction of its surface. The vertical lines were drawn to the surfaces of different sheet tilting from the same point and the different vertical lines were drawn from the same point, distributed in a fan-shape, with the decrease of the inclined angles of the sheet from left to right, when the inclined angles of the sheet increased gradually, which was shown in Figure 10. That is to say, the node flow law obtained by the numerical simulation in this paper was consistent with the above-mentioned research.

## 4. Forming Experiment

**Figure 11:**
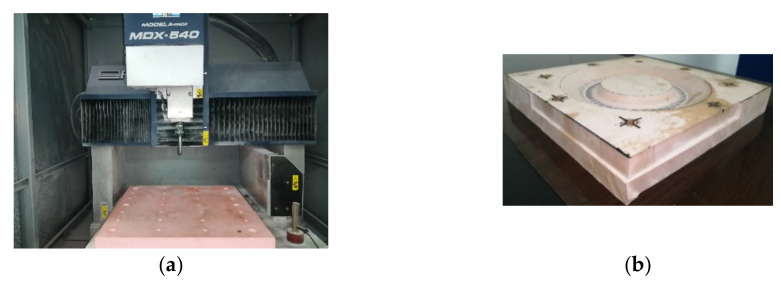
The forming experimental setup: (**a**) the 3-axis CNC machining center and (**b**) the fabricated support.

In order to study the specific influence of sheet tilting, the model shown in Figure 1 was taken as the research object and the forming experiments were carried out four times with the inclined angles of $15^{\circ }$, $20^{\circ }$, $25^{\circ }$, and $30^{\circ }$, respectively. In the incremental forming experiments, the forming was conducted on the three-axis CNC machining center, shown in Figure 11a; the 1060 Aluminum sheet with the thickness of 0.88 mm was taken as the forming sheet and the support was milled with chemical wood, as shown in Figure 11b; the forming tool was a hemispherical tool head with a diameter of 10 mm that was made of W6Mo5Cr4V2 high-speed steel. The values of the spindle speed and feed rate were 400 rpm and 600 mm/min, respectively.

**Figure 12:**
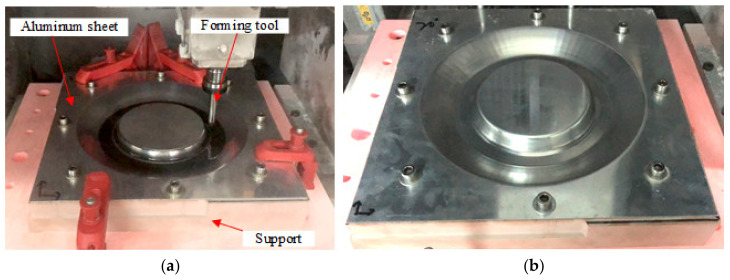
The forming experiment process: (**a**) the forming process and (**b**) the formed sheet metal part.

Figure 12a,b show the experimental process of CNC incremental forming and the formed sheet metal part under a $15^{\circ }$ inclined angle of sheet, respectively.

**Figure 13:**
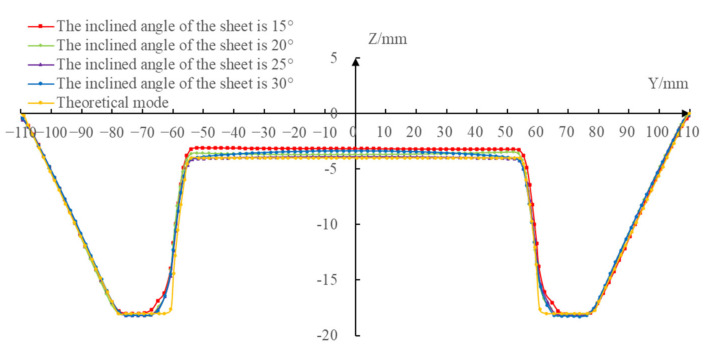
The profile curves of the X = 0 section.

**Figure 14:**
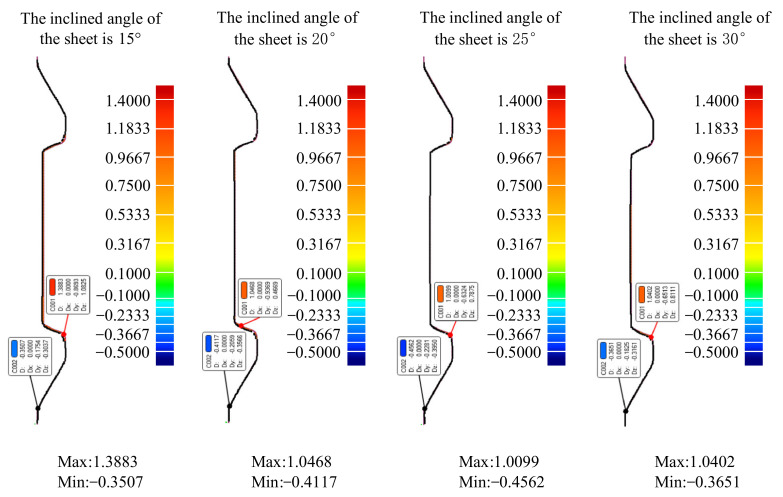
The normal deviation of the profile.

In order to compare the influence of the sheet postures on the profile accuracy of the formed parts under the different inclined angles of the sheet postures, the *X* = 0 section (the coordinate system was defined as shown in Figure 1) profiles of the four formed parts were measured at 2 mm intervals by utilizing CMM (a three coordinate measuring instrument) to get the point cloud. The 3D distribution map and contour distribution curve were drawn by the corresponding software and compared with the theoretical contour, then the different contour accuracy could be obtained. Figure 13 shows the profile curves that were made using the Excel software. The Geomagic Studio/Qualify software was used to evaluate the normal deviation of the *X* = 0 section profiles between the formed parts and the theoretical model, which is shown in Figure 14. 

It can be seen from the profile comparison curves shown in Figure 13 that the profile curves of the formed parts under the different inclined angles of the sheet were consistent with the theoretical profile curves and the difference among the profile curves was relatively small; there was a subtle difference, which was mainly reflected in the bottom of the difficult-forming surface. The larger the inclined angle of the sheet was, the closer the bottom profile curve was to the theoretical profile curve.

According to the normal profile deviation data shown in Figure 14, the maximum positive deviations between the profile curves of the formed parts and the theoretical profile curve on the *X* = 0 section were 1.3883 mm, 1.0468 mm, 1.0099 mm, and 1.0402 mm, respectively, and the maximum negative deviations were 0.3507 mm, 0.4117 mm, 0.4562 mm, and 0.3651 mm, respectively, when the inclined angles of the sheet were $15^{\circ }$, $20^{\circ }$, $25^{\circ }$, and $30^{\circ }$, respectively. The results showed that the maximum positive deviations and the maximum negative deviations under different inclined angles of the sheet occurred on the difficult-forming surface and the easy-forming surface, respectively. From the profile comparison curves and profile normal deviation data, the profile curves of the formed parts were in good agreement with the theoretical profile curves under different inclined angles of the sheet in the whole forming area, i.e., the inclined angle of the sheet had little effect on the profile accuracy of the formed parts. 

**Figure 15:**
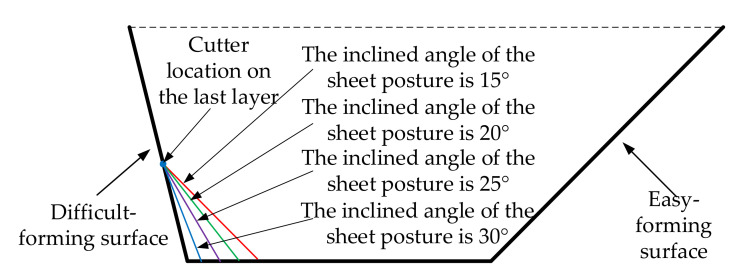
The sheet postures for the difficult-forming surface.

The reason for the subtle difference in the profile curves at the bottom of the difficult-forming surface under the different inclined angles of the sheet was shown in Figure 15. The whole forming process was close to the end when the forming tool was moved to the cutter location points of the last layer. The larger the inclined angle of sheet was, the closer the surface of sheet at the cutter location points of the last layer was to the difficult-forming surface. Therefore, the larger the inclined angle of the sheet was, the more consistent the bottom of the difficult-forming surface of the formed part was with the theoretical model. 

**Figure 16:**
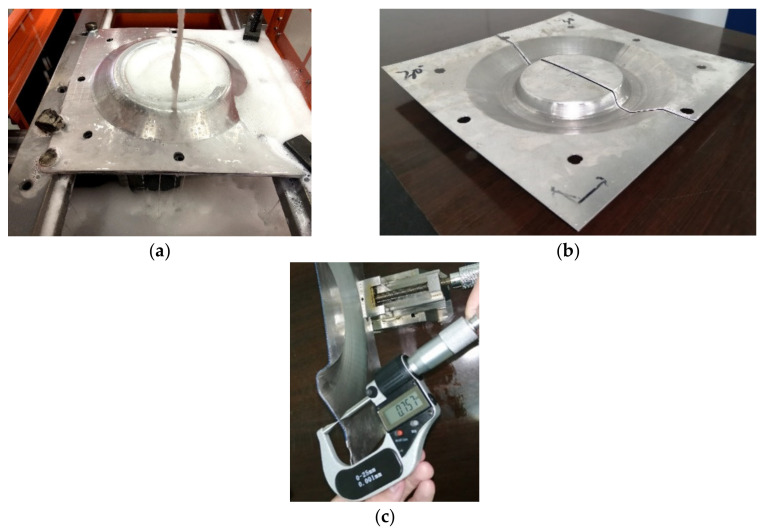
Thickness measurement: (**a**) wire cutting, (**b**) part after cutting, and (**c**) measuring.

**Figure 17:**
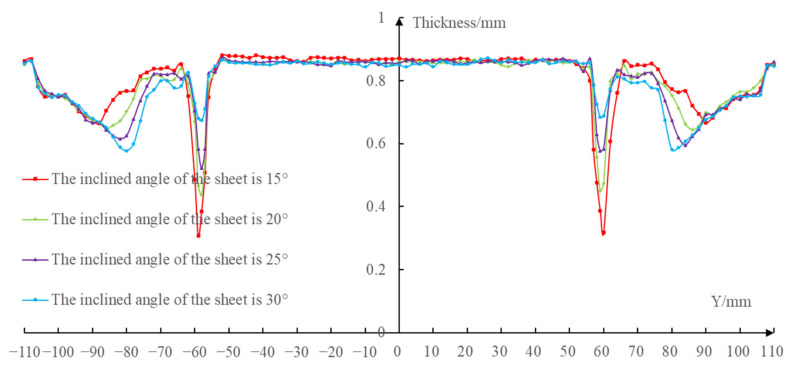
The thickness distribution curves.

In order to measure the thickness of the formed part, the wire cutting machine was used to cut the formed part along the *X* = 0 section to get two cutting parts (Figure 16a,b). Then, the double pointed micrometer was used to measure the thickness along the *X* = 0 section at an interval of 2 mm (Figure 16c) and the measured data were imported into the Excel software to draw the thickness distribution curves. Figure 17 shows the thickness distribution comparison curves at the *X* = 0 section under different inclined angles of the sheet postures.

It can be seen from the thickness distribution curves that the thickness of the easy-forming surface (−110 mm≤Y≤-78 mm and 78 mm≤Y≤110 mm) decreased gradually, while the thickness of the difficult-forming surface (−61 mm≤Y≤-55mm and 55 mm≤Y≤61 mm) increased gradually with the increase of the inclined angles of the sheet. In addition, the minimum thickness of the formed parts occurred on the difficult-forming surface when the inclined angles of the sheet were $15^{\circ }$, $20^{\circ }$, $25^{\circ }$; however, the minimum thickness of the formed part occurred at the bottom of the easy-forming surface when the inclined angle of the sheet was $30^{\circ }$. This phenomenon was consistent with the results of the finite element numerical simulation.

The reason is that the forming angles on the easy-forming surface increased gradually and the forming angles on the difficult-forming surface decreased gradually with the increase of the inclined angles of the sheet. The forming angles on the easy-forming surface were far greater than that of the difficult-forming surface when the inclined angle of the sheet reached $30^{\circ }$. With the continuous thinning of the sheet metal, the minimum thickness finally appeared at the bottom of the easy-forming surface.

## 5. Conclusions

The influence of the inclined angle of the sheet on the overall profile accuracy of the formed part is small in the single point CNC incremental forming. The difference is mainly reflected in the bottom of the difficult-forming surface. The larger the inclined angle of the sheet is, the closer the bottom profile curve of the difficult-forming surface is to the theoretical profile curve. The inclined angle of the sheet has a great influence on the material flow, plastic strain, and thickness distribution of the formed parts. In the whole forming area, there are obvious differences in the node flow curves, although the distribution curves of the nodes under the different inclined angles of the sheet are consistent. The flow curves of the same node under the different inclined angles of the sheet are fan-shaped. In addition, the plastic strain and thickness on the easy-forming surface increase with the increase of the inclined angle of the sheet, while the plastic strain and thickness on the difficult-forming surface decrease with the increase of the inclined angle of the sheet.

The problem can be solved by adjusting the inclination of the sheet. However, the influence of sheet tilting on thickness thinning is different according to the different tilting range. Therefore, the key is to determine the inclination angle of the sheet reasonably. This paper studied the influence of the different inclinations of the sheet on the forming quality in CNC incremental forming. The results of the studies can help choose the inclination angle of the sheet reasonably and realize the non-fracture forming of the difficult forming surface with a large forming angle.

## Figures and Tables

**Table 1:** Mechanical property parameters.

Name	Density /kg•m^−3^	Elastic Modulus /GPa	Poisson Ratio	Yield Stress /MPa	Tangent Modulus /GPa	Hardening Coefficient
Al1060	2700	55.94	0.324	153.6	2.9	0.19775
GCr15	8160	218	0.30	—	—	—
W6Mo5Cr4V2	7810	212	0.29	—	—	—

**Table 2:** Forming angle change table.

Name	Horizontal Sheet	The Inclined Angle of the Sheet Is $15^{\circ }$	The Inclined Angle of the Sheet Is $20^{\circ }$	The Inclined Angle of the Sheet Is $25^{\circ }$	The Inclined Angle of the Sheet Is $30^{\circ }$
Forming angle of the easy-forming surface.	$30^{\circ }$	$45^{\circ }$	$50^{\circ }$	$55^{\circ }$	$60^{\circ }$
Maximum forming angle of the difficult-forming surface.	$75^{\circ }$	$60^{\circ }$	$55^{\circ }$	$50^{\circ }$	$45^{\circ }$

## Data Availability

Not applicable.
